# Implementation of a model of awareness-raising for taxi motorcyclists in Benin in relation to helmet use: a quasi-experimental study

**DOI:** 10.1186/s12889-022-13857-8

**Published:** 2022-07-26

**Authors:** Bella Hounkpe Dos Santos, Alphonse Kpozehouen, Yolaine Glele Ahanhanzo, Donatien Daddah, Emmanuel Lagarde, Yves Coppieters

**Affiliations:** 1 Ecole de Santé Publique, Université libre de Bruxelles, Brussels, Belgium; 2 Institut Régional de Santé Publique, Université d’Abomey-Calavi, Ouidah, Benin; 3 Université de Bordeaux, Bordeaux, France

**Keywords:** Quasi-experimental, Awareness, Helmet, Road accident, Effectiveness

## Abstract

**Background:**

This study aims to test the effectiveness of an awareness-raising model designed based on the theory of planned behaviour regarding helmet use for motorcycle taxi drivers.

**Methods:**

This quasi-experimental study took place in the cities of Parakou (intervention group) and Porto Novo (control group). Over a three-month period, a package of awareness-raising activities, based on the theory of planned behaviour, have been implemented in the intervention area. Data relate to knowledge, attitudes and practices regarding helmet use was collected prospectively before the intervention, at the end, and 6 months later. Stata 15 was used for data analysis. Chi-square or Fisher, Student’s or Kruskal-Wallis tests was carried out. The difference-in-difference method was used to determine the specific effect of the awareness activities.

**Results:**

After the intervention, there was an improvement in the total score in both groups compared to baseline. The total score increased by 0.2 (0.06–0.3) in the experimental group when the number of sessions attended increased by one (*p* = 0.005). The difference-in-difference estimator measured among subjects who attended at least one awareness session, controlling for socio-demographic variables, showed a significantly higher difference in the total score of subjects in the experimental group compared to those in the control group both at the end of the interactive sessions and 6 months later.

**Conclusion:**

This model improves the helmet-wearing behaviour of motorbike taxi drivers in the experimental area. It could be adapted and applied to other socio-professional groups and other types of users.

## Background

Road accidents are a major public health problem across the world. They are the leading cause of death for young people aged 15 to 29. Apart from the high number of deaths in the economically active population, these accidents also cause disabilities and represent a heavy economic burden for families and countries. Low-income countries account for around 13% of road deaths [1]. This burden is very high in Africa [2, 3]. In most African countries, the use of vehicles that do not meet key safety standards, the dilapidated state of road infrastructure, and the absence, inadequacy or insufficient enforcement of road safety laws continue to expose road users to fatal road accidents [4–6]. Added to this are the behaviours of road users. One of the main risk factors for road accidents and related trauma is the attitudes and behaviours of users, most notably: speeding; driving under the influence of alcohol or any other psychoactive drug; not wearing a helmet, seatbelt or child restraint; and distracted driving, such as using a mobile phone [1, 7–11]. Despite these well-known factors, superstitious drivers are more likely to attribute accidents to fate [12, 13]. Although aware of the protection offered by helmets, many motorcycle drivers and passengers do not wear one [14, 15]. This situation is all the more worrying, since the most vulnerable road users, such as pedestrians, cyclists and motorcyclists, account for more than half of all road deaths in the African sub-region, according to the World Health Organization (WHO). This figure is an underestimate, due to the poor quality of the data provided by the countries in the region, especially when we consider the rising number of motorcycles and journeys by motorcycle in these countries, which is contributing to the increase in road accidents [1, 16]. Accidents cause motorcyclists more limb injuries than head injuries, but the latter are responsible for almost half of all deaths [9]. Authors found that wearing helmets reduced the risk of head trauma, severe trauma, hospitalisations and death [17–19]. Similarly, in his cross-sectional study, Singleton argues that skull fractures, brain contusions and intracranial haemorrhages were significantly less common among helmeted motorcyclists injured in road crashes than among those not wearing a helmet [20].

In Benin, young people aged 20 to 40 are the group most frequently involved in road accidents. They also account for nearly half of all victims injured or killed in such accidents. In addition, motorcycles are involved in more than half of all accidents, and their drivers or passengers represent more than half of the fatalities (CNSR, 2017). In Benin, motorcycles are the main means of travel for road users. The proportion of households that owns a motorcycle continues to grow, rising in 10 years from less than 45% in 2001 to more than 55% in 2011 [21]. Motorcycle taxi drivers are among those who travel mainly by motorcycle, using this means of transport as a taxi to carry passengers. This mode of transport is mostly used for trips within cities. These motorcycle taxi drivers do not always perceive the risks associated with their profession [22].

According to the WHO and several authors, in low- and middle-income countries, only an approach integrating user behaviour and several other interventions will be able to prevent trauma and death from road accidents in a cost-effective manner [1, 6, 23–26]. The main effective interventions are legislative reforms accompanied by political will, and implementing measures [1, 23, 25], such as awareness-raising and education of the population [27], and increased police control [1, 24]. Concerning specifically the wearing of helmets, the implementation of helmet legislation seems to be effective in increasing the use of helmets, and reducing head injuries and deaths from road accidents [28–30], even more so if it is accompanied by public awareness and education, which affect user knowledge and attitudes towards helmet-wearing behaviour [6, 27, 31]. User knowledge is defined as the state of knowing about helmet wearing, and attitude is understood as users’ subjective judgement, specifically their beliefs about the likely consequences of wearing a helmet [32]. To ensure behavioural change in individuals, it is necessary to implement educational interventions based on proven theories or models [31–33]. According to the theory of planned behaviour (TPB), behaviour is determined by intention, which is the conscious decision to take a certain action. It is guided by a combination of three considerations: attitude, the subjective norm, and the perception of control over behaviour. According to this theory, attitude is the set of people’s beliefs regarding the consequences of the said behaviour, multiplied by the evaluation of those consequences. These are the judgments about the desirability of the behaviour and its consequences. The subjective norm is an individual’s set of normative beliefs, and his or her motivation to comply with the standards. It is therefore the perceived social pressure to conform or not conform to the behaviour, the considerations of influence, and the opinion of relatives on the behaviour. Perceived behavioural control is the perceived ease or difficulty of performing a given behaviour: the belief in one’s ability to succeed in the targeted behaviour. In addition, environmental, demographic and personal factors influence all three types of beliefs [31–34].

Benin adopted the law on compulsory helmet wearing for motorcycle drivers and passengers in April 1972, but it was not accompanied by enforcement measures. It was not until 2014 that this law began to be effectively implemented for motorcycle drivers, with mass awareness-raising, police controls and penalties. It is clear, however, that there are still drivers who do not always wear helmets, especially in certain localities of the country. How effective would a helmet awareness programme for motorcycle taxi drivers in Benin be? Would such a programme help to reduce cases of road accident-related traumatic brain injury within this target group? This study aims to test the effectiveness of an awareness model to improve the helmet-wearing behaviour of motorcycle taxi drivers and to help reduce the risk of traumatic brain injury among this target group.

## Methods

### Study framework

**Fig. 1:**
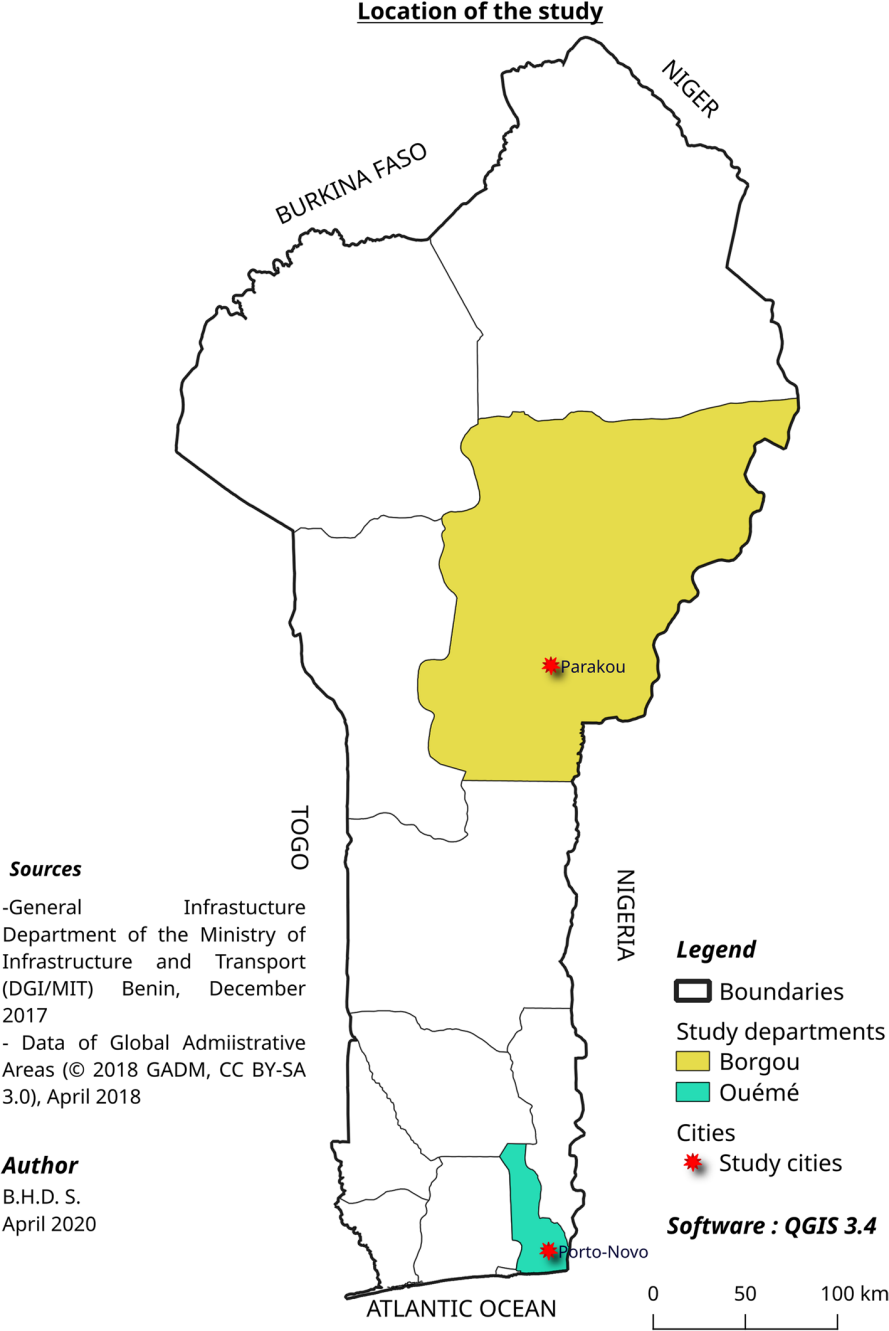
Location of the study. Departments and administrative boundaries of Benin. Study departments are marked in different colours. Red stars represent study cities. Data sources: DGI/MIT Benin and GADM.org. Copyright holder: BHDS [35]

The study took place in two cities in Benin: Porto Novo and Parakou (Fig. 1). To identify the study cities, we took into account the fact that these two cities are the second (Porto Novo) and third (Parakou) largest in the country. In both cities, legislation concerning the wearing of helmets is enforced, but not always consistently. They are also located in departments that are at the two extremes of the country (north and south), reducing the risk of control group contamination (Fig. 1). In these two cities, as in the rest of the country, motorcycle taxi drivers are organised in unions of motorcycle taxi drivers. The experiment was implemented among motorcycle taxi drivers in parks in Parakou (intervention group), while those in Porto Novo did not benefit from the awareness activity package and were the control group.

### Type of study

**Fig. 2:**
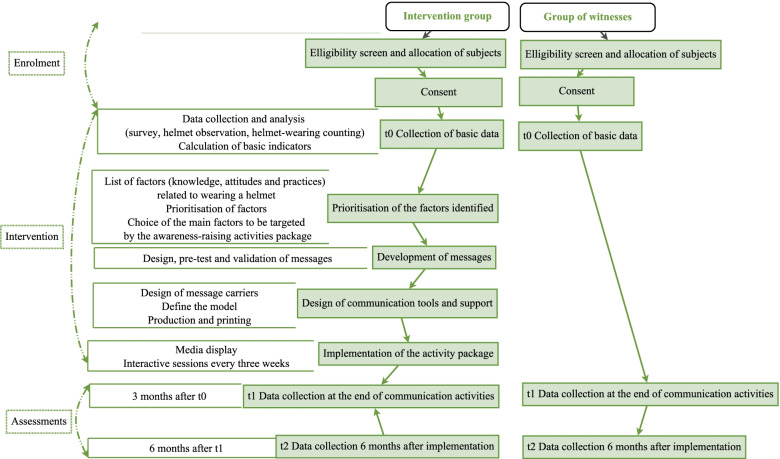
Overview of the study. In the green background are the different stages of the study in each group, and in the white background, the main activities at each stage and the timing. Software Dia [35]

It was a quasi-experimental study that used control groups, pre-tests and post-test [36], which was conducted with motorcycle taxi drivers. Figure 2 shows an overview of the study scheme.

### Targets and inclusion criteria

The targets of the study were two groups of motorcycle taxi drivers from Parakou and Porto Novo. The motorcycle taxi drivers in the Parakou group received the awareness activity package, and those from Porto-Novo did not. To be included in this study, motorcycle taxi drivers must be at least 18 years old, had frequented one of the selected parks regularly for at least 3 months, drived a motorcycle taxi as a main and daily activity, and been willing to participate in the study.

### Sampling and sample size

Sampling was done at two stages. In each city, the list of the main parks was obtained from the town hall. Two parks were chosen at random from the parks in each city. In each park, the drivers were informed in collaboration with the park managers. Within the parks, convenience sampling was used. All drivers who meet the inclusion criteria were recruited.

The minimum sample size calculated was 42 for each zone (intervention, non-intervention) but taking into account the continuity correction and a lost-to-follow-up rate 83 people was recruited in experimental group and 60 in control group.

### Intervention

This was the implementation of a package of awareness-raising activities in the intervention area, preceded by a series of preparatory activities, such as prioritising key factors, developing messages, and designing tools (Fig. 2). This package supplemented the helmet-wearing controls, penalties and mass awareness activities carried out in both areas.

**Fig. 3:**
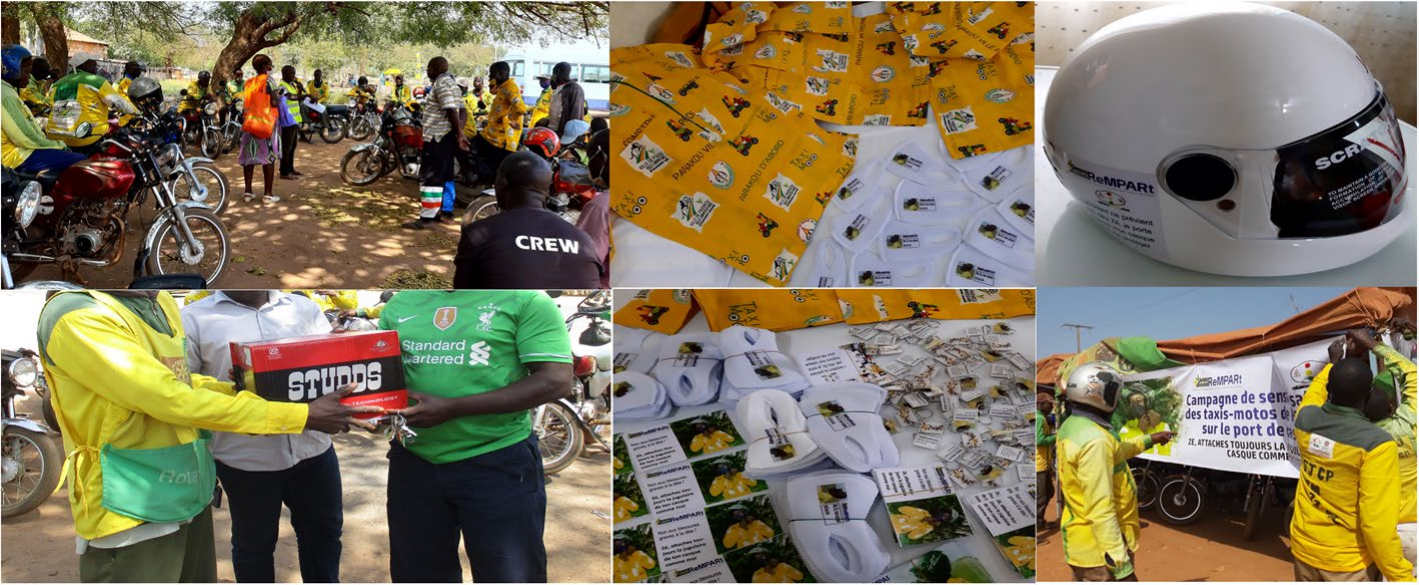
Photos of implementation. From top to bottom and from left to right: an interactive session, gift giving to a motorcycle taxi driver by the chiefs of park, motorcycle taxi uniforms, mufflers, keyrings, stickers and helmets with awareness-raising messages, setting up of a banner

The implementation of the awareness-raising activities package involved local communication in the intervention area with interactive awareness sessions on helmet wearing for drivers, and the dissemination of messages through other channels such as banners, stickers for motorcycles, mufflers, keyrings, helmets, motorcycle taxi uniforms, video spots, text messages and directs calls (Fig. 3).

### Data collection

Data were collected prospectively, before the implementation of the activities, at the end, and 6 months later. The same data were collected as in the baseline collection, using the same tools. Data collection tools was a questionnaire that was designed based on the TPB. The data collected related to [35]:

General Information;

Socio-demographic data (age, sex, marital status, ethnicity, religion, level of education, average income, number of dependents);

History (how long they have been driving motorcycles and in the motorcycle taxi profession, whether they own their motorcycle, road accidents, number of days of driving per week, average number of hours of driving per day, sanctions for not wearing a helmet);

Knowledge: Five (5) questions (advantages, disadvantages, characteristics of a quality helmet);

Attitudes: Eight (8) questions (perception, judgement related to wearing a helmet);

Subjective norms: Four (4) questions (influence of those around you);

Perceived behavioural control: Three (3) questions (perceived constraints in relation to wearing a helmet);

Intention of wearing a helmet: Four (4) questions (possession of a helmet and reason for purchase, willingness to wear helmet);

Practices of use, and information on the helmet: Six (6) questions (frequency, time/period of wearing of the helmet, mode of use, type and condition of the helmet).

### Data processing and analysis

The data collected via KoboCollect were extracted and processed using Excel and Stata 15 software. They were analysed using Stata 15.

When analysing the baseline data, the study population was described according to their socio-demographic data and the number of interactive sessions in which they had participated. The subjects included in the initial data collection, but who do not respond to the other collections, were compared with the respondents in order to verify the existence of a bias. These comparisons were made using the Chi^2^ test after checking that the conditions were met (the expected values ≥5). If the conditions were not met, we used Fisher’s exact test [35].

The actual data analysis compared subjects not excluded from the intervention group with those from the control group. An overall score for level of knowledge, attitude and practice was be calculated for each individual. This overall score was obtained from the scores of the different groups of variables (knowledge, attitudes and practices). Scores were calculated by assigning points to each response given by the enrolled subject. The total points was calculated to keep the score for each group of variables. These scores varied as follows, by group of variable: knowledge (0 to 14), attitudes (0 to 24), subjective norms (2 to 13), perceived behaviour control (0 to 1), intention (1 to 17) and practices (0 to 28).

Average scores was calculated by zone (intervention and control) for each collection. Comparisons was made between the mean scores of pre- and post-awareness, intervention and control areas, and according to socio-demographic characteristics. Student’s statistical test was used for these comparisons. For these tests, the equality of variances was tested using the robust Levenne’s test for variance of equality. If this test is significant, the Hartley test (S2max/S2min < 3) was performed [35].

After this preliminary analysis, the difference-in-difference (DD) estimator, an approach using a linear parametric model, was used [37–39] to determine the specific effect of the awareness-raising activities in order to assess whether these have brought any added value. This estimator was the difference in mean overall score in the intervention group before and after the awareness-raising activities, from which the same difference is subtracted in the control group. It corresponds to the coefficient β_3_ of the regression equation *Y*_*i*_ = *β*_0_ + *β*_1_*T*_*i*_ + *β*_2_*t*_*i*_ + *β*_3_(*T*_*i*_ ∗ *t*_*i*_) + *λX*_*it*_ + *ε*_*i*_ in which *Y*_*i*_ was the overall score of the subjects, *T*_*i*_ the groups (intervention and control), *t*_*i*_ the period (pre- and post-intervention), *X*_*it*_ the variables related to the socio-demographic characteristics and background of the subjects, and *ε*_*i*_ the random error [35].

The significance level of the statistical tests was 5%.

## Results

### Cohort retention

**Fig. 4:**
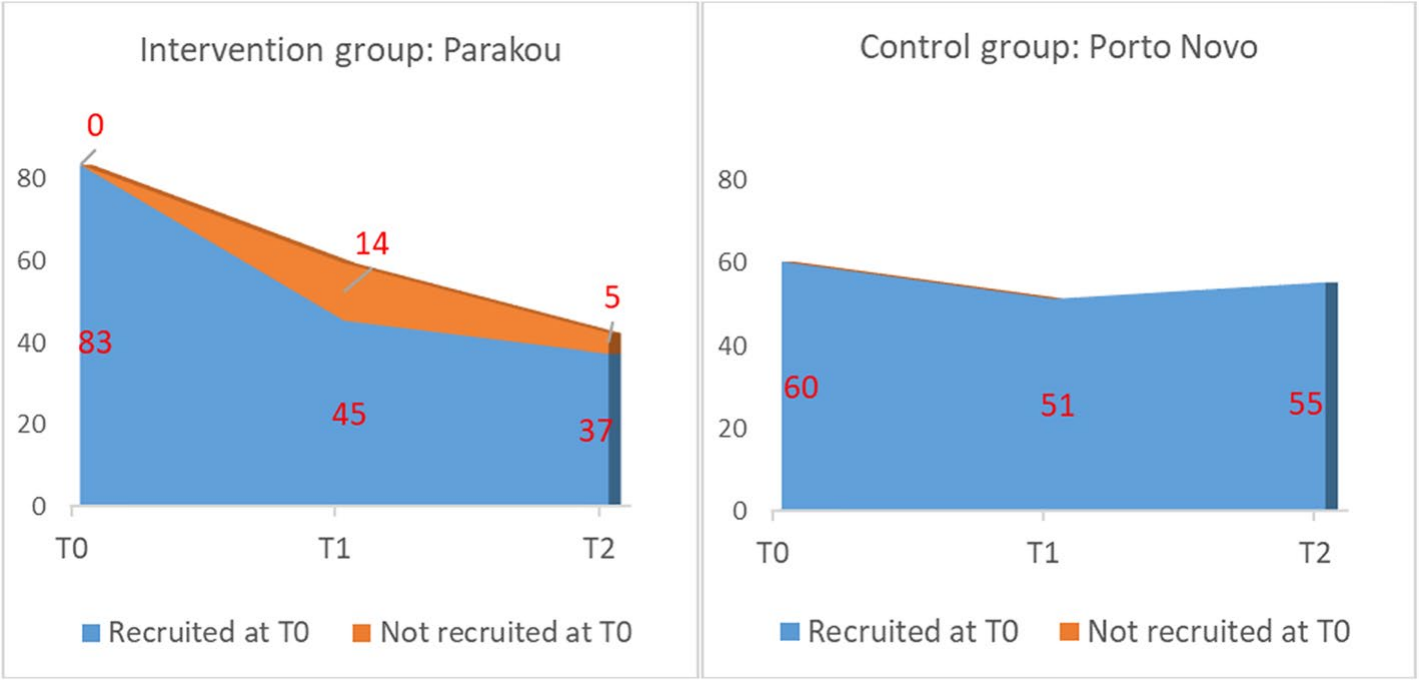
Number of subjects who participated in each collection

Cohort retention, especially in the quasi-experimental zone, was difficult despite the arrangements made to ensure the participation of enrolled subjects in the awareness sessions and data collection. Figure 4 shows the number of subjects surveyed at each collection.

A comparison was made between the initial enrolees who did not participate in subsequent rounds and the rest of the cohort to see if they had any specific characteristics that could lead to bias. There was no difference between the two groups (Table 1).

**Table 1:** Comparison of respondents and non-respondents, motorcycle taxi drivers included in a quasi-experimental study, Benin, 2021

*Variables*	Respondents (*n* = 124) % or mean (SD)	No-respondents (*n* = 33) % or mean (SD)	*p*-value
Marital status			0.792
*Single*	8.9	6.1	
*Married or engaged*	90.3	93.9	
*Divorced or widowed*	0.8	0.0	
Educational level			0.536
*None*	33.1	30.3	
*Primary*	33.9	21.2	
*Secondary*	24.2	36.4	
*University*	8.9	12.1	
Average daily income			0.604
*< 1500*	91.8	97.0	
*1500–5000*	7.3	3.0	
*5000–10,000*	1.0	0.0	
Number of dependants			0.681
*< = 3 persons*	22.6	27.3	
*4 to 6 persons*	36.3	36.4	
*7 persons and more*	41.1	36.4	
Length of time in the profession			0.154
*< 1 year*	4.8	6.1	
*1–4 years*	28.2	15.1	
*5–9 years*	21.0	12.1	
*> = 10 years*	46.0	66.7	
Owner of the motorbike			0.114
*Yes*	75.0	87.9	
*No*	25.0	12.1	
Accident history			0.878
*Never*	52.4	54.6	
*Once*	29.8	24.2	
*2–3 time*	14.5	18.2	
*4 time and more*	3.2	3.0	
Age	39.2 (11.0)	43.4 (13.2)	0.053

### Socio-demographic characteristics of subjects

All motorbike taxi drivers were male in both areas. There was no difference between the two groups in terms of age, length of service and average daily income. However, compared to drivers in the control area, those in the intervention group were more often single and more likely to own the motorbike. They had a higher level of education and fewer dependants and were more likely to have a history of road accidents (Table 2).

**Table 2:** Socio-demographic characteristics of subjects, motorcycle taxi drivers included in a quasi-experimental study, Benin, 2021

*Variables*	Intervention group (*n* = 97) % or mean (SD)	Control group (*n* = 60) % or mean (SD)	*p*-value
Sex			
*Man*	100	100	
Marital status			0.006
*Single*	13.4	0.0	
*Married or engaged*	86.6	98.3	
*Divorced or widowed*	0.0	1.7	
Educational level			<0.001
*None*	18.6	55.0	
*Primary*	26.8	38.3	
*Secondary*	39.2	6.6	
*University*	15.5		
Average daily income			0.604
*< 1500*	2.1	1.7	
*1500–5000*	88.7	93.3	
*5000–10,000*	9.3	5.0	
Number of dependants			0.037
*< = 3 persons*	30.9	11.7	
*4 to 6 persons*	34.0	40.0	
*7 persons and more*	35.1	48.3	
Length of time in the profession			0.916
*< 1 year*	5.2	5.0	
*1–4 years*	26.8	23.3	
*5–9 years*	17.5	21.7	
*> = 10 years*	50.5	50.0	
Owner of the motorbike			0.000
*Yes*	92.8	53.3	
*No*	7.2	46.7	
Accident history			0.035
*Never*	44.3	66.7	
*Once*	36.1	16.7	
*2–3 time*	16.5	13.3	
*4 time and more*	3.1	3.3	
Age	41.2 (10.2)	39.1 (12.4)	0.275

### Follow-up to the outreach sessions

The total number of outreach sessions initially planned was four, with one 3-to-5-hour session every 3 weeks. At the time of implementation, during discussions with the motorbike taxi drivers, they preferred the interactive sessions to be held every fortnight and to last 2 h. Seven interactive sessions were then conducted. The median number of sessions attended by the participants was one session. However, 31 subjects (31.9%) attended three or more sessions.

### Evolution of scores in the groups

Prior to the implementation of the intervention, subjects in the experimental group had higher levels of knowledge and attitudes (9.4 and 18.7 respectively) than those in the control group (7.7 and 17.4). In addition, their overall score on helmet-wearing behaviour was also better than the control group (68.1 vs. 64.3). However, despite this better level in the experimental group, the perceived behavioural control component was slightly improved at baseline for the control group, which had a score of 0.7 versus 0.0 (Table 3). This score increased to 0.9 at the collections following the implementation of the communications package in the experimental group, with a difference that was no longer significant compared to the control group, which had scores of 1.

**Table 3:** Changes in scores by group and time period, motorcycle taxi drivers included in a quasi-experimental study, Benin, 2021

*Scores*	T0			T1			T2		
	Intervention (*n* = 83)	Control (n = 60)	*p*-value	Intervention (*n* = 59)	Control (*n* = 51)	*p*-value	Intervention (*n* = 42)	Control (*n* = 55)	*p*-value
	Mean (SD)			Mean (SD)			Mean (SD)		
Knowledge	9.4 (2.1)	7.7 (1.7)	<0.001	9.5 (1.8)	7.9 (2.2)	<0.001	9.3 (1.9)	7.4 (2.1)	<0.001
Attitude	18.7 (1.9)	17.4 (2.4)	0.001	19.7 (1.6)	19.2 (1.5)	0.104	20.0 (1.4)	18.2 (1.6)	<0.001
Subjective norms	8.5 (2.3)	7.9 (2.0)	0.140	9.4 (2.2o)	8.6 (2.3)	0.081	9.7 (2.0)	9.8 (2.4)	0.834
Perceived behavioural control	0.0 (0.0)	0.1 (0.3)	0.017	0.9 (0.2)	1.0 (0.0)	0.103	0.9 (0.2)	1.0 (0.1)	0.412
Intention	14.2 (2.1)	14.7 (1.1)	0.134	16.0 (1.6)	16.1 (1.0)	0.753	16.1 (1.5)	16.1 (1.2)	0.945
Helmet use behaviour	17.4 (2.2)	16.5 (3.0)	0.062	18.6 (3.1)	16.0 (2.9)	<0.001	19.9 (1.9)	16.1 (2.0)	<0.001
Total score	68.1 (5.7)	64.3 (4.6)	<0.001	74.2 (4.6)	68.8 (5.0)	<0.001	75.8 (4.1)	68.4 (5.8)	<0.001

After the intervention, there was an improvement in the total score in both groups compared to baseline. This gain was observed both immediately after the interactive sessions and 6 months later. It was higher in the experimental group, which maintained, and increased, the gap with the control group. Thus, the overall score in the experimental group was 68.1 at T0, 74.2 at T1 and 75.8 at T2 compared to 64.3, 68.8 and 68.4 respectively in the control group. In the experimental group, after the implementation of the communications package, the scores improved for all groups of variables apart from the level of knowledge, but the practice of wearing a helmet was much higher compared to the control group (Table 3).

It should be noted that in the experimental group, the overall level of helmet-wearing behaviour among motorbike taxi drivers improved with the number of interactive sessions attended. Thus, the total score increased by 0.2 (0.06–0.3) with each session (*p* = 0.005).

### Contribution to helmet-use behaviour change

To assess the effectiveness of the intervention, scores were measured in the experimental group as a whole and then among the subjects who had participated in at least one awareness-raising session. The comparisons were made with the control group using the difference-in-difference method, which takes into account the scores in the two groups and their evolution over time, while adjusting for socio-demographic variables.

Table 4 shows that by not taking into account participation in the interactive sessions, the intervention was not effective immediately after the implementation of the interactive sessions, as the difference in total knowledge, attitude and practice scores relating to helmet use between the experimental and control groups was not significant (*p* = 0.210), but 6 months later this difference became significant (*p* = 0.007).

**Table 4:** Results of difference-in-difference estimates taking into account covariates without taking into account participation in interactive sessions, motorcycle taxi drivers included in a quasi-experimental study, Benin, 2021

*Total score*	Intervention		Control		Diff (I-C)	p-value
	Effective	Mean	Effective	Mean		
T0	83	68.4	60	65.0	3.4	0.001
T1	59	75.2	51	70.2	5.0	<0.001
Diff-in-Diff T0-T1					1.6	0.210
T0	83	69.0	60	65.8	3.2	0.002
T2	42	76.1	55	69.0	7.1	<0.001
Diff-in-Diff T0-T2					3.9	0.007

As shown in the Table 5, when only those subjects in the experimental group who had attended awareness sessions were considered, the effectiveness of the intervention was observed both after the implementation of the interactive sessions (diff-in-diff T0-T1 = 3.4) and 6 months later (diff-in-diff T0-T2 = 5.4) with a significant difference-in-difference.

**Table 5:** Results of difference-in-difference estimates taking into account the covariates while taking into account participation in at least one interactive session, motorcycle taxi drivers included in a quasi-experimental study, Benin, 2021

*Total score*	Intervention		Control		Diff (I-C)	p-value
	Effective	Mean	Effective	Mean		
T0	49	70.2	60	68.0	2.2	0.043
T1	54	78.8	51	73.2	5.6	<0.001
Diff-in-Diff T0-T1					3.4	0.011
T0	49	73.8	60	71.7	2.1	0.064
T2	40	81.8	55	74.3	7.5	<0.001
Diff-in-Diff T0-T2					5.4	<0.001

## Discussion

The present study aims to assess the effectiveness of a model of helmet awareness among motorbike taxi drivers in Benin, based on the theory of planned behaviour. The experiment showed a significant improvement in the total score in the experimental group both immediately after the interactive sessions and 6 months later. In addition, the more subjects participated in the interactive sessions, the higher their total score. These results demonstrate that the implementation of the awareness-raising package improved the helmet-wearing behaviour of motorbike taxi drivers in the experimental area. Similar results have been obtained by authors among teenage students in the UK [32] and cement workers in Iran [31],), i.e., improved helmet-wearing behaviour following education based on the theory of planned behaviour. A study of student pre-drivers regarding compliance with traffic laws based on the same theory proved effective, with significant improvement in scores after the intervention [40]. The maintenance of learning observed 6 months after the experiment in our study was also observed in the United Kingdom at the five-month follow-up [32]. Poulter et al., on the other hand, did not observe the maintenance of these scores 5 months after the intervention [40].

The significant difference in total score noted immediately after the implementation of the intervention between the two groups (T1) could be attributed to participation in the interactive sessions. The persistence of a significant score difference 6 months after the end of the interactive sessions (T2) could be explained by the fact that drivers had continued to be exposed to the messages through the banners left at the parks, the video ads that continued to circulate and the awareness products distributed.

In the present study, after the implementation of the intervention, there was an increase in all scores among the experimental group compared to the control group, but only the practice score went from a non-significant difference to a significant difference. This observation could be explained by other road safety activities outside the experiment, such as mass awareness-raising, police controls and penalties. Thus, there was no significant difference between the intervention and control groups with respect to subjective norm scores, perceived behavioural control and intention. This differs from Quine et al. who found that the behavioural, normative and control beliefs and intentions of intervention participants became more positive than those of control participants, and that the effect was maintained over time Jafaralilou et al. found that the experimental group had significant improvements in all scores: helmet use, attitude, subjective norm, behavioural control and intention [31].

An improvement in perceived behavioural control was observed in both groups. For Ali et al. this was the strongest predictor of intention to wear a helmet, followed by subjective norm and attitude [41].

Limitations:

The high attrition in the experimental group is a limitation in this study. The enrolment of new subjects at the second collection made it possible to obtain a minimum size for the analyses, especially at T2.

The subjects included in this study by convenience sampling, may not be representative of the overall population of motorcycle taxi drivers. In addition, not all the motorcycle taxi drivers included in the intervention group reached with all the interactives sessions.

## Conclusion

As research in the field of road accident prevention is rare in Benin, this study help fill a significant gap. It provide factual data on the rate of helmet use among motorcycle taxi drivers and on their knowledge, attitudes and practices (KAP) relating to helmet use. The study shows the effectiveness of awareness raising activities targeting specific groups and based on proven theories such as TPB.

In order to induce the behaviour of permanent helmet wearing by motorbike taxi drivers, this intervention model could be replicated among several motorbike taxi groups. It could also be adapted and applied to other socio-professional groups of two-wheeled users or even other types of users.

## Data Availability

The datasets generated and/or analysed during the current study are not publicly available as it is the property of the ReMPARt project which financially supported the implementation of the awareness-raising model, but are available from the corresponding author on reasonable request.

## Abbreviations

CNSR: National Centre for Road Safety
DD: Difference in Difference
KAP: Knowledge, Attitudes and Practices
TPB: Theory of planned behaviour
WHO: World Health Organization
